# Autophagy Markers Are Altered in Alzheimer’s Disease, Dementia with Lewy Bodies and Frontotemporal Dementia

**DOI:** 10.3390/ijms25021125

**Published:** 2024-01-17

**Authors:** Antonio Longobardi, Marcella Catania, Andrea Geviti, Erika Salvi, Elena Rita Vecchi, Sonia Bellini, Claudia Saraceno, Roland Nicsanu, Rosanna Squitti, Giuliano Binetti, Giuseppe Di Fede, Roberta Ghidoni

**Affiliations:** 1 Molecular Markers Laboratory, IRCCS Istituto Centro San Giovanni di Dio Fatebenefratelli, 25125 Brescia, Italy; sbellini@fatebenefratelli.eu (S.B.); csaraceno@fatebenefratelli.eu (C.S.); rnicsanu@fatebenefratelli.eu (R.N.); rsquitti@fatebenefratelli.eu (R.S.); rghidoni@fatebenefratelli.eu (R.G.); 2 Neurology 5/Neuropathology Unit, Fondazione IRCCS Istituto Neurologico Carlo Besta, 20133 Milan, Italy; marcella.catania@istituto-besta.it (M.C.); elena.vecchi@unimi.it (E.R.V.); giuseppe.difede@istituto-besta.it (G.D.F.); 3 Service of Statistics, IRCCS Istituto Centro San Giovanni di Dio Fatebenefratelli, 25125 Brescia, Italy; ageviti@fatebenefratelli.eu; 4 Neuroalgology Unit, Fondazione IRCCS Istituto Neurologico Carlo Besta, 20133 Milan, Italy; erika.salvi@istituto-besta.it; 5 Data Science Center, Fondazione IRCCS Istituto Neurologico Carlo Besta, 20133 Milan, Italy; 6 Dipartimento di Scienze di Laboratorio, Ospedale Isola Tiberina-Gemelli Isola, 00186 Rome, Italy; 7 MAC-Memory Clinic and Molecular Markers Laboratory, IRCCS Istituto Centro San Giovanni di Dio Fatebenefratelli, 25125 Brescia, Italy; gbinetti@fatebenefratelli.eu

**Keywords:** Alzheimer’s disease, dementia with Lewy bodies, frontotemporal dementia, neurodegeneration, autophagy, ATG5, UBQLN2, ULK1, LC3, brain

## Abstract

The accumulation of protein aggregates defines distinct, yet overlapping pathologies such as Alzheimer’s disease (AD), dementia with Lewy bodies (DLB), and frontotemporal dementia (FTD). In this study, we investigated ATG5, UBQLN2, ULK1, and LC3 concentrations in 66 brain specimens and 120 plasma samples from AD, DLB, FTD, and control subjects (CTRL). Protein concentration was measured with ELISA kits in temporal, frontal, and occipital cortex specimens of 32 AD, 10 DLB, 10 FTD, and 14 CTRL, and in plasma samples of 30 AD, 30 DLB, 30 FTD, and 30 CTRL. We found alterations in ATG5, UBQLN2, ULK1, and LC3 levels in patients; ATG5 and UBQLN2 levels were decreased in both brain specimens and plasma samples of patients compared to those of the CTRL, while LC3 levels were increased in the frontal cortex of DLB and FTD patients. In this study, we demonstrate alterations in different steps related to ATG5, UBQLN2, and LC3 autophagy pathways in DLB and FTD patients. Molecular alterations in the autophagic processes could play a role in a shared pathway involved in the pathogenesis of neurodegeneration, supporting the hypothesis of a common molecular mechanism underlying major neurodegenerative dementias and suggesting different potential therapeutic targets in the autophagy pathway for these disorders.

## 1. Introduction

The abnormal accumulation of misfolded and aggregated protease-resistant proteins is a shared feature among major neurodegenerative disorders [1]. In the three most common neurodegenerative dementias, Alzheimer’s disease (AD), dementia with Lewy bodies (DLB), and frontotemporal dementia (FTD), the accumulation of protein aggregates defines distinct, yet overlapping pathologies. AD is the most common neurodegenerative dementia and is characterized by intra- and extra-cellular amyloid-β (Aβ) peptide aggregates forming amyloid plaques, and by the intracellular accumulation of hyperphosphorylated tau protein polymerized into paired helical filaments, forming neurofibrillary tangles [2,3]. DLB is characterized by the presence of intraneuronal inclusions called Lewy bodies, with α-synuclein as their main component [4], also sharing neuropathological features with AD, such as the presence of amyloid plaques [5]. FTD is a neuropathologically heterogeneous neurodegenerative disorder characterized by the presence in the brain of different proteins deposits, i.e., tau, ubiquitin, fused-in-sarcoma (FUS) protein, TAR DNA-binding protein 43 (TDP-43), and dipeptide repeat proteins in *C9orf72* pathological expansion carriers, forming distinct inclusion bodies [6,7].

The accumulation of protein aggregates in AD, DLB, and FTD suggests that dysfunctions in the protein homeostasis network might underlie the pathology in all these disorders. Autophagy is an evolutionarily conserved pathway aimed at delivering cytoplasmic cargo to lysosomes for degradation, essential for proteostasis in the nervous system, and specifically for the degradation of irreversibly misfolded or aggregated proteins [8,9]. Macroautophagy (also referred to as autophagy) is the most known form of autophagy and it involves the compartmentalization of cytoplasmic cargoes into vesicles called autophagosomes, which fuse with lysosomes for cargoes degradation by lysosomal hydrolases [10]. In contrast, chaperone-mediated autophagy (CMA) directly delivers substrates into lysosomes, although only proteins with a specific motif (the KFERQ-like motif) are recognized as CMA substrates [11]. Pathogenic proteins accumulated in neurodegenerative disorders are substrates of both macroautophagy and CMA pathways. The ubiquitin-proteasome system parallels and integrates macroautophagy by tagging with ubiquitin, a highly conserved 76-residue polypeptide, molecules destined to become degraded by the proteasome, a big cytoplasmatic proteolytic complex [12].

In recent years, several studies focused their attention on the role of autophagy in neurodegeneration, investigating disruptions in the pathway that could be related to the pathogenesis and treatment of neurodegenerative diseases [13,14]. Accordingly, the involvement of autophagy in neurodegenerative dementias is supported by the presence of typically mutated genes that are required for the proper functioning of the autophagy and lysosomal pathways: the *PSEN1* gene encoding presenilin 1, mutated in AD, which is a lysosomal ATPase; the *SNCA* gene encoding α-synuclein, mutated in Parkinson’s disease (PD)/DLB, which is involved in autophagosome biogenesis and transport, CMA, and lysosomal integrity; the *MAPT* gene encoding microtubule-associated protein tau, the *GRN* gene encoding progranulin, and the *C9orf72* gene encoding C9orf72, mutated in FTD, which are involved in autophagosome biogenesis and transport, CMA, lysosomal integrity, and homeostasis. In addition, AD, DLB, and FTD pathogenic proteins impair autophagy pathways, specifically by disrupting autophagosome biogenesis and transport, vesicular trafficking, and lysosomal pathways [15]. Recently, in a genetic study, we reported that genes controlling key endo-lysosomal processes, such as protein sorting/transport and lysosomal enzymatic activity regulation, might be involved in AD, DLB, and FTLD pathogenesis, suggesting a cross-disease mechanism behind these pathologies [16].

The analysis of several autophagy markers, involved in macroautophagy and CMA, has drawn increasing attention considering the opportunity to gain important insights into the different steps of the autophagic pathway involved in neurodegeneration. Microtubule-associated protein 1A/1B-light chain 3 (LC3) is a soluble protein distributed ubiquitously in mammalian tissues and cells able to initiate macroautophagy. A cytosolic form of LC3 (LC3-I) is conjugated to phosphatidylethanolamine to form LC3-phosphatidylethanolamine conjugate (LC3-II), recruited to autophagosomal membranes and activating autophagosome generation [17,18]. LC3 can also be conjugated to Rab5^+^, clathrin^+^ endosomes containing Aβ in a process of LC3-associated endocytosis (LANDO) [19]. In a murine model of AD, LANDO was described to enable the removal of Aβ in microglia and ameliorate their pathology [19]. Autophagy-related protein 5 (ATG5), a member of the ATG family, initiates the autophagy process [20]. ATG5 binds ATG12 and ATG16L1 resulting in an E3-like ligase complex, necessary for the autophagosome expansion and lipidation of LC3 [21]. *Atg5* overexpression led to neuroprotection in injured motoneurons [22] while the deletion of *Atg5* led to a dramatic increase in Aβ plaque number and size within the hippocampus of 5xFAD mice [19]. UNC-51-like kinase-1 (ULK1) plays a central role in starvation-induced autophagy, integrating signals from the upstream to the downstream central autophagy pathway [23]. Upon starvation, ULK1 dissociates from the mechanistic target of rapamycin complex 1 (mTORC1), activating ULK1 kinase activity [24]. Alterations in ULK1 were observed in AD brain specimens, which correlates with clinical dementia progression [25]. Ubiquilin-2 (UBQLN2) protein is involved in the cross-talk between the ubiquitin-proteasome system and CMA [26]. It is mainly involved in protein homeostasis, directing misfolded or redundant proteins towards the proteasome to become degraded [27]. Mutations in the *UBQLN2* gene were demonstrated to cause neurodegeneration in the FTD/ALS spectrum [28,29,30].

In the current study, we sought to investigate the levels of ATG5, UBQLN2, ULK1, and LC3 proteins in different brain regions (the temporal, frontal, and occipital cortex) and in the plasma of patients affected by AD, DLB, and FTD, to identify common mechanisms.

## 2. Results

### 2.1. Subjects’ Demographic Characteristics

The demographic characteristics of 66 brain specimens and 120 plasma samples are shown in Table 1. Considering brain specimens, ages were significantly different between phenotype groups (*p* = 0.0004), in particular comparing control subjects (CTRL) vs. DLB (*p*_adj_ = 0.002) and vs. FTD (*p*_adj_ = 0.002) patients, with a similar trend, vs. AD (*p*_adj_ = 0.059) patients. No significant differences were observed for gender. Considering plasma samples, ages were significantly different between phenotype groups (*p* < 0.001), in particular comparing DLB vs. CTRL (*p*_adj_ < 0.001), vs. FTD (*p*_adj_ < 0.001), and vs. AD (*p*_adj_ = 0.032). Gender was significantly different among phenotype groups as well (*p* = 0.040).

### 2.2. Autophagy Pathway Is Altered in Brain

**Figure 1:**
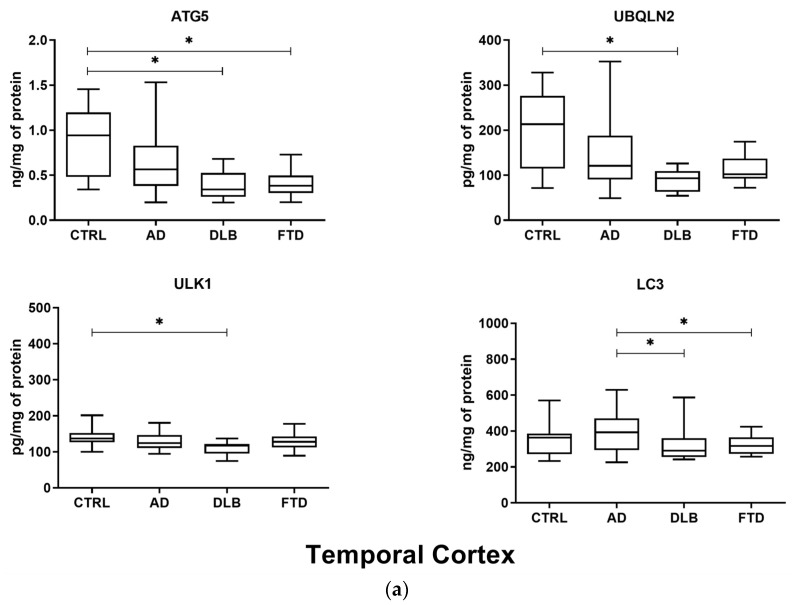

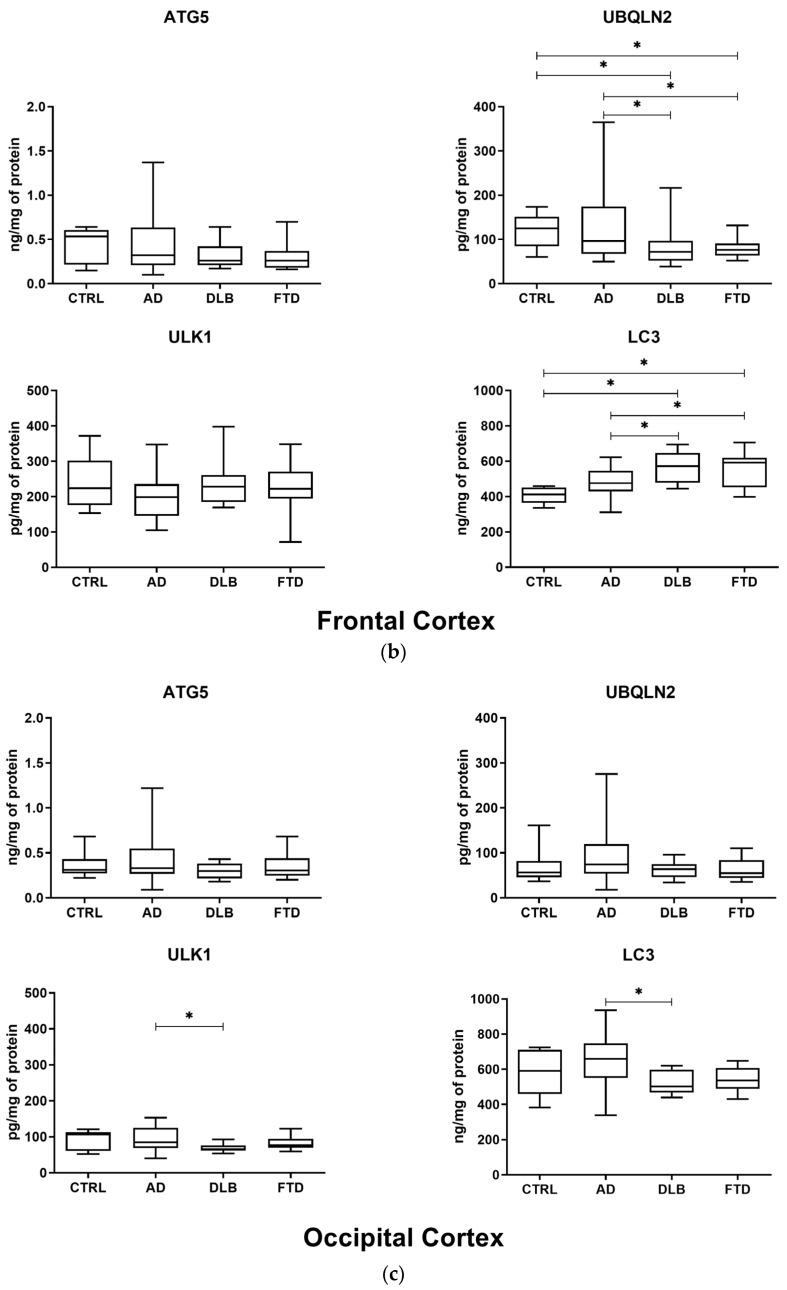
Boxplots of ATG5, UBQLN2, ULK1, and LC3 levels in brain specimens. (**a**) Temporal cortex. A statistically significant decrease in ATG5 levels was observed in DLB and FTD groups compared to those in the CTRL group. UBQLN2 and ULK1 levels resulted in being significantly decreased in the DLB group compared to those in the CTRL group. LC3 was decreased in DLB and FTD specimens compared to that in AD specimens. (**b**) Frontal cortex. UBQLN2 levels were significantly decreased in DLB and FTD groups compared to those in the CTRL group. LC3 levels resulted in being significantly increased in the DLB and FTD groups compared to those in the CTRL group. UBQLN2 was decreased in the DLB and FTD groups compared to that in the AD group. LC3 was increased in DLB and FTD specimens compared to that in AD specimens. (**c**) Occipital cortex. No differences were shown between patient groups and the CTRL group in terms of ATG5, UBQLN2, ULK1, and LC3 levels. ULK1 and LC3 were decreased in the DLB group compared to those in the AD group. * *p*_adj_ < 0.05. Linear regression analysis corrected for age as a confounder was conducted, with Benjamini–Hochberg correction for multiple comparisons.

Levels of ATG5, UBQLN2, ULK1, and LC3 were analyzed in brain specimens of 32 AD, 10 FTD, and 10 DLB patients, and 14 non-demented CTRL subjects. A significant decrease of ATG5 levels was found in temporal cortex of DLB and FTD patients compared to CTRL subjects (DLB vs. CTRL *p*_adj_ = 0.013; FTD vs. CTRL *p*_adj_ = 0.020) (Figure 1a). A similar non-significant trend was shown in the temporal cortex of AD patients (Figure 1a) and in the frontal cortex of all patient groups (Figure 1b). UBQLN2 resulted in being decreased in DLB patients compared to that in CTRL subjects in temporal cortex specimens (*p*_adj_ = 0.013), along with a similar non-significant trend in AD and FTD groups (Figure 1a). UBQLN2 was also decreased in the frontal cortex of DLB and FTD brains (DLB vs. CTRL *p*_adj_ = 0.034; FTD vs. CTRL *p*_adj_ = 0.034), with a similar non-significant trend in AD specimens (Figure 1b). Moreover, UBQLN2 was decreased in the frontal cortex of DLB and FTD specimens compared to AD specimens (DLB vs. AD *p*_adj_ = 0.030; FTD vs. AD *p*_adj_ = 0.030). ULK1 was decreased in DLB temporal cortexes compared to those of the CTRL (*p*_adj_ = 0.030) (Figure 1a) and in the DLB occipital cortex compared to that in AD patients (*p*_adj_ = 0.045) (Figure 1c). LC3 resulted in being increased in the frontal cortex of DLB and FTD patients compared to that in CTRL subjects (DLB vs. CTRL *p*_adj_ = 0.013; FTD vs. CTRL *p*_adj_ = 0.013) (Figure 1b). No differences between the patient groups and CTRL were shown in occipital cortex specimens for ATG5, UBQLN2, ULK1, and LC3 levels (Figure 1c). However, LC3 was decreased in temporal (*p*_adj_ = 0.036) and occipital (*p*_adj_ = 0.033) cortexes of DLB patients compared to those in AD patients, and in the temporal cortex of FTD patients compared to that in AD patients (*p*_adj_ = 0.041). LC3 was increased in the frontal cortex of DLB and FTD patients compared to that of AD patients (DLB vs. AD *p*_adj_ = 0.020; FTD vs. AD *p*_adj_ = 0.013).

### 2.3. ATG5 and UBQLN2 Concentrations Are Decreased in Plasma

**Figure 2:**
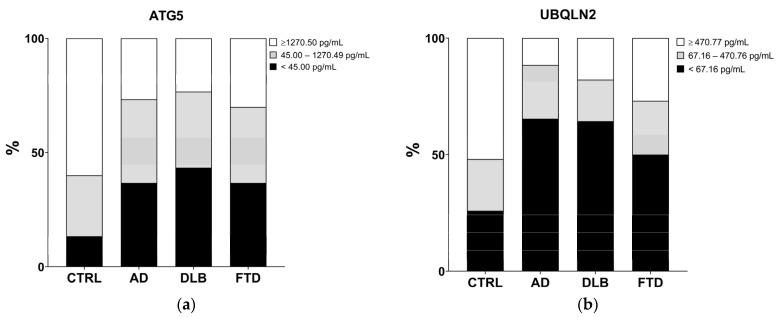
Concentrations of ATG5 (**a**) and UBQLN2 (**b**) in plasma samples. Levels of ATG5 resulted in being decreased in AD, DLB, and FTD samples compared to those in CTRL samples (**a**). Levels of UBQLN2 resulted in being decreased in AD samples compared to those in CTRL samples, showing a downward trend in DLB and FTD samples (**b**). Multinomial logistic regression corrected for age and gender as confounders was performed, with Benjamini–Hochberg correction for multiple comparisons.

Decreased plasma levels of ATG5 and UBQLN2 were observed in the pathological groups compared to those in the CTRL (Figure 2). Specifically, plasma samples with an ATG5 concentration of <45.00 pg/mL (the minimum detected value) compared to that of samples with an ATG5 concentration of ≥1270.50 pg/mL (median value of the detected values) had a higher probability of belonging to the AD, DLB, or FTD group with respect to the CTRL group (AD vs. CTRL *p*_adj_ = 0.024; odds ratio (OR) = 7.26; 95% confidence interval (CI) = 1.68–31.31; DLB vs. CTRL *p*_adj_ = 0.027; OR = 12.91; 95% CI = 2.53–65.98; FTD vs. CTRL *p*_adj_ = 0.030; OR = 6.57; 95% CI = 1.55–27.80) (Figure 2a). Plasma samples with a concentration of UBQLN2 of <67.16 pg/mL (the minimum detected value) compared to samples with UBQLN2 concentrations of ≥470.77 pg/mL (median value of the detected values) had a higher probability of belonging to the AD group than the CTRL group. The same not statistically significant downward trend was shown for DLB and FTD groups compared to the CTRL group (AD vs. CTRL *p*_adj_ = 0.048; OR = 9.88; 95% CI = 2.06–47.31; DLB vs. CTRL *p*_adj_ = 0.078; OR = 5.24; 95% CI = 1.12–24.43; FTD vs. CTRL *p*_adj_ = 0.078; OR = 4.19; 95% CI = 1.08–16.29) (Figure 2b). No differences were observed among the patient groups for both ATG5 and UBQLN2 concentrations. All comparisons were adjusted for age and gender; only in the DLB group plasma concentrations were influenced by these confounders. LC3 and ULK1 levels were not detectable in all plasma samples.

## 3. Discussion

Autophagy represents one of the several pathways accountable for delivering intracellular cargo to lysosomes for degradation. Dysfunctions in this complex pathway may be a major mechanism behind neurodegenerative diseases, given their critical role in the clearance of aggregated protein, a main common feature in the pathogenesis of AD, DLB, and FTD [14]. The presence of several mutations and variants in genes involved in autophagy and endo-lysosomal pathways found in neurodegeneration supports the vision that alterations in these pathways could contribute to the pathogenesis of neurodegenerative diseases [14,16]. It has been recently suggested that autophagic activity declines with age in diverse organisms: studies in *Caenorhabditis elegans*, rodents and human cells reported an age-dependent reduction in lysosomal proteolytic function that impairs autophagic flux [31,32,33,34]. In particular, an age-associated reduction was reported in ATG5 levels in mice brain tissue and in *ATG5* expression in humans [35,36], while *Atg* overexpression extends the lifespan in Drosophila and mice [37,38,39]. Mutant UBQLN2^P497H^ accumulation was also associated with an age-dependent decrease in several core autophagy-related proteins [27]. An aging effect on autophagy markers was also reported in cervical motor neurons of aged mice with increased LC3 levels [40].

In the current study, we demonstrate that the autophagy pathway is altered in AD, DLB, and FTD. In particular, we measured altered levels of proteins involved in autophagosome formation, ATG5 and LC3, and in the regulation of the ubiquitin-proteasome system, UBQLN2. Specifically, ATG5 concentrations resulted in being significantly decreased in the temporal cortex of DLB and FTD patients, showing only a downward trend in the AD group, and in the frontal cortex specimens of all patients, with heterogenous values in AD patients. In plasma, the ATG5 concentration was significantly decreased in all patients compared to that in the CTRL group. A reduction in ATG5 protein appears to be a systematic feature shared among the major neurodegenerative dementia groups investigated, supporting the hypothesis that a defective initiation of autophagy may represent a common mechanism. Accordingly, previous findings showed lower values of ATG5 in serum/plasma samples of AD, mild cognitive impairment (MCI), mixed dementia, and PD subjects [41,42], and a downward trend of ATG5 concentrations in the temporal cortex of AD and DLB patients compared to those in CTRL subjects [43]. However, the literature is still poor and controversial in terms of autophagy protein levels since other studies showed increased plasma levels of ATG5 in MCI and dementia patients [44]; these controversial results may be due to the heterogeneity of the analyzed cohorts.

UBQLN2 levels were reduced in both the plasma samples and temporal/frontal cortex of patients compared to those in CTRL subjects. In particular, UBQLN2 concentrations resulted in being significantly reduced in AD plasma samples, showing a downward trend in DLB and FTD compared to CTRL subjects. The differences in brain levels resulted in being statistically significant in DLB temporal cortexes and in DLB/FTD frontal cortexes compared to those of CTRL subjects. While UBQLN2 has been investigated in relation to the presence of mutations in the *UBQLN2* gene associated with amyotrophic lateral sclerosis (ALS)/FTD [45], the number of studies on sporadic FTD is still small. Previous studies showed ubiquilin immunoreactivity in cytoplasmic and nuclear inclusions in synucleinopathies [46] and provided evidence that *UBQLN2* dysregulation may contribute to α-synuclein-mediated toxicity [47]. Moreover, UBQLN2 levels were reported as decreased in the temporal cortex of AD patients, but not co-localized with neurons bearing hyperphosphorylated tau or tau inclusions [48]. It is possible that the loss of UBQLN2 contributes to AD by interfering with Aβ-related pathology as well, but this hypothesis needs to be supported by further investigation. Furthermore, it has been reported that the loss of UBQLN2 reduces the expression of an ATPase subunit pump, critical for the regulation of vacuolar acidification and the maturation of autophagosomes [49]. These last findings may explain the involvement of UBQLN2 in the three distinct forms of dementia.

ULK1 was significantly reduced in DLB temporal cortex specimens compared to that in CTRL subjects, suggesting an impairment of upstream autophagy that might occur in the DLB disease process, mainly in the temporal cortex. In a cellular model of Lewy bodies disease, it was reported that upstream autophagy-related proteins, including ULK1, were localized in Lewy bodies, and their expression increased in parallel with the accumulation of inclusions; however, an analysis of total brain lysates in PD and DLB subjects did not show alterations in ULK1 levels, supporting the hypothesis that an impairment of upstream autophagy might occur in the pathogenesis of Lewy body disease with a resultingly altered autophagy process in an advanced stage of the disease [50].

Levels of LC3 resulted in being significantly increased in DLB and FTD frontal cortex specimens compared to those in CTRL specimens, showing a similar trend in the temporal, frontal, and occipital cortex of AD specimens. The increase in LC3 concentration in the DLB frontal cortex compared to that CTRL is in agreement with previous evidence showing an increase in LC3-II in DLB brain specimens and in transgenic mice overexpressing mutant α-synuclein. This supports the role of LC3 in macroautophagy induction to remove α-synuclein accumulation [51]. Accordingly, a recent study found that the overexpression of human *MAPT* in animal models leads to a deficit in autophagy by repressing autophagosome–lysosome fusion. The resultingly increased levels of LC3-II, measured in both transgenic mice and AD human brains, were correlated with an autophagosome accumulation that confirmed the involvement of LC3 in autophagy induction by the presence of protein aggregation in neurodegenerative disease [52]. In line with previous studies reporting an exacerbation of Aβ accumulation in the brain tissue of LANDO-deficient mice [19], the trend of an increase in LC3 levels in AD brains may suggest the reactive involvement of autophagy in AD pathology.

Levels of LC3 and ULK1, not detectable in plasma samples, represent a limitation of this study; more sensitive assays might allow for future studies in this direction. Furthermore, the absence of statistical significance between groups in ATG5 frontal cortex analysis, even though they follow the same resulting downward trend in the temporal cortex, may be due to the small size of the sample.

Our findings support the hypothesis of the differential involvement of distinct autophagy-related pathways (i.e., macroautophagy, mitophagy, and aggrephagy) in different forms of dementias, as well as in different brain areas [53]. In particular, the decreased levels of ATG5 in brain specimens and plasma samples could be related to the impairment of ATG5-mediated canonical autophagy in neurodegenerative disorders. Many studies have demonstrated Aβ and APP degradation via the upregulation of macroautophagy, even if other authors reported the opposite [54]. A loss of ATG proteins results in the accumulation of ubiquitin-positive protein aggregates and interferes with the formation and fusion of autophagosomes with lysosomes, having an impact on autophagy initiation [43,55,56]; these alterations could be involved in neurodegenerative diseases [57,58]. In accordance with our results, the suppression of ubiquilin and ATG5 protein expression, and therefore autophagy initiation, is associated with an increase in the immature form of LC3 and a lack of LC3-I to LC3-II conversion in the brain [56,59]. Of note, besides the ubiquitin-like protein systems ATG5–ATG12, ATG7–ATG8, ATG16, and ATG9, autophagosome formation can be activated by other complexes such as ULK1 and PI3K-Beclin 1-VPS34 [60,61]. This could explain the accumulation of enlarged and atypical LC3-II-positive vesicles and their colocalization with protein involved in neurodegenerative diseases observed in brain specimens [43,51,52]. It is also known that ubiquitin-independent mitophagy receptors use LC3 to mediate mitophagy [62]. The high levels of LC3 observed in our study could be explained by an attempt to reinforce ubiquitin-independent mitophagy to prevent mitochondrial damage along the course of the disease. However, future studies could confirm this hypothesis.

Furthermore, recent findings demonstrated that the levels of autophagy and lysosomal degradation proteins are increased with long-term memory stimulation and that the lack of autophagy impairs learning and memory [63,64]. The accumulation of protein and the deficit in stimulus-dependent regulations, as cognitive impairments found in AD, DLB, and FTD, imply that these dysregulations could be related to the alterations in the autophagy process.

The findings of our study on brain specimens, especially in AD cases, are consistent with the differential involvement of autophagy in distinct brain areas, with some findings being significantly different in brains with dementia compared to those in CTRL brains and others showing only a trend that is not statistically relevant. We hypothesize that these results are related to the neuropathological heterogeneity of primary degenerative dementias across brain tissue and to the inter-individual variability of neuropathological changes [65,66,67].

In conclusion, our study is in agreement with the hypothesis of common molecular mechanisms underlying major neurodegenerative dementias, supported by the defects in ATG5- and UBQLN2-related autophagy processes and the autophagy stimulation linked to increased levels of LC3 detected in patients affected by neurodegeneration. Molecular alterations in the autophagic pathway could play a role in a shared pathway involved in the pathogenesis of neurodegenerative dementias, supporting the possibility of employing modulators of different steps in the autophagy pathway as potential therapeutics in these disorders.

## 4. Materials and Methods

### 4.1. Subjects

Brain specimens from 66 subjects, i.e., *n* = 32 AD, *n* = 10 FTD, and *n* = 10 DLB patients, and *n* = 14 non-demented CTRL subjects were analyzed. The tissues were obtained from the biobanks of Neurology 5/Neuropathology Unit, IRCCS Besta, Milan and Institut D’Investigacions Biomediques August Pi I Sunyer (IDIBAPS), Barcelona. The diagnosis was confirmed via a neuropathological assessment according to current criteria [68,69,70]. All the brain specimens were processed and analyzed at IRCCS Besta.

Human plasma samples from 120 subjects, i.e., *n* = 30 AD, *n* = 30 DLB, and *n* = 30 FTD sporadic patients, and from *n* = 30 subjects with normal cognitive function, comprising the CTRL group, were included for the plasma study. Samples were obtained from the biobanks of IRCCS Fatebenefratelli, Brescia and Neurology 5/Neuropathology Unit, IRCCS Besta, Milan. Patients considered for plasma study underwent clinical and neurological examination at the MAC-Memory Clinic IRCCS Fatebenefratelli, Brescia, and at the Neurology 5/Neuropathology Unit, IRCCS Besta, Milan. Clinical diagnoses were made in accordance with international guidelines [71,72,73,74,75]. Patients provided written informed consent. The study protocol was approved by the local ethics committee (Prot. N. 94/2018).

### 4.2. Biochemical Analyses

Brain specimens from temporal, frontal, and occipital cortexes were homogenized in 9 volumes of Lysis buffer (100 mM NaCl, 10 mM EDTA, 0.5% NP40, 0.5% Sodium Deoxycholate, 10 mM Tris-HCl pH 7.4), to which was added Complete Protease Inhibitor Cocktail (Roche, Basel, Switzerland), sonicated for 30 s using an ultrasonic homogenizer (Sonopuls, Bandelin, Berlin, Germany), and centrifuged at 800× *g*, 4 °C for 5 min. The supernatant was collected, stored at −80 °C, and further used for biochemical studies. The total protein concentration in human brain homogenates was assessed using a Pierce BCA Protein Assay kit (Thermo Scientific, Rockford, IL, USA). ELISA assays were used for the measurement of ATG5, UBQLN2, LC3 (ELISAGenie, Dublin, Ireland), and ULK1 (Nordic BioSite AB, Taby, Sweden) levels. Each specimen was analyzed in duplicate, following the manufacturer’s protocol. ATG5, UBQLN2, ULK1, and LC3 concentrations were normalized to total protein concentration and expressed in weight/mg of protein.

Plasma concentrations of human ATG5 and UBQLN2 proteins were determined using ELISA kits (ELISAGenie, Dublin, Ireland); the ULK1 plasma concentration was measured with human ULK1 ELISA Kit (Nordic BioSite AB, Taby, Sweden); and the LC3 plasma concentration was measured using an LC3 ELISA kit (Cusabio Biotech Co., Ltd., Wuhan, China). The assays were performed following the manufacturer’s protocol. All samples were analyzed in duplicate.

### 4.3. Statistical Analysis

For demographic variables, the normality assumption of age as a continuous variable was evaluated with a Shapiro–Wilk test. One-way ANOVA, with Bonferroni’s post hoc correction, was used for a comparison across the four subject groups of the normally distributed continuous variables. A Kruskal–Wallis test with Dunn’s post hoc tests was used for the group comparisons of non-normally distributed variables. A chi-square test was used to analyze differences in gender distribution among the study groups. For the analysis of ATG5, UBQLN2, ULK1, and LC3 levels in brain homogenates, a normality assumption of continuous variables was evaluated with a Shapiro–Wilk test. For non-normally distributed variables, we applied logarithmic transformation to the dependent variable. Linear regression models were used to model the relationship between quantitative levels of ATG5, UBQLN2, ULK1, and LC3 in brain homogenates and phenotype groups. Levels of the categorical phenotype variable (AD, DLB, FTD, and CTRL) were compared to each other. Benjamini–Hochberg correction for multiple testing was applied. Regression analyses were corrected for age as a covariate.

For analysis in the plasma, the association between the protein concentration (for ATG5 and UBQLN2) and the four subject groups was assessed via multinomial logistic regression, where the concentration figured as the independent variable with three levels (1: values < 45.00 pg/mL for ATG5 or <67.16 pg/mL for UBQLN2; 2: values from 45.00 to 1270.49 pg/mL for ATG5 or from 67.16 to 470.76 pg/mL for UBQLN2; 3: values ≥ 1270.50 pg/mL for ATG5 or ≥470.77 pg/mL for UBQLN2). Moreover, we controlled for the confounders age and gender. Benjamini–Hochberg correction for multiple testing was applied. The analysis was performed using R (version 4.1.2), STATA 11, and SPSS software (version 29), and significance was set at 0.05 after correction.

## Figures and Tables

**Table 1:** Demographic characteristics of patients and controls.

**Brain Specimens**					
	**CTRL (*n* = 14)**	**AD (*n* = 32)**	**DLB (*n* = 10)**	**FTD (*n* = 10)**	***p*-Value**
Male/Female, *n* (%)	5/9 (35.7%/64.3%)	19/13 (59.4%/40.6%)	5/5 (50.0%/50.0%)	7/3 (70.0%/30.0%)	0.129 ^a^
Age, years	61.2 ± 13.3	69.6 ± 11.6	77.8 ± 3.6	78.7 ± 3.5	<0.001 ^b^
**Plasma Samples**					
	**CTRL (*n* = 30)**	**AD (*n* = 30)**	**DLB (*n* = 30)**	**FTD (*n* = 30)**	***p*-Value**
Male/Female, *n* (%)	7/23 (23.3%/76.6%)	9/21 (30.0%/70.0%)	17/13 (56.6%/43.3%)	13/17 (43.3%/56.6%)	0.040 ^a^
Age, years	65.2 ± 11.2	69.0 ± 7.8	75.4 ± 6.9	65.8 ± 8.5	<0.001 ^c^

CTRL, controls; AD, Alzheimer’s disease patients; DLB, dementia with Lewy bodies patients; FTD, frontotemporal dementia patients; ^a^ chi-squared test; ^b^ Kruskal–Wallis with Dunn’s post hoc tests; ^c^ one-way ANOVA test with Bonferroni’s post hoc correction. Means ± standard deviation.

## Data Availability

The data presented in this study are available in the Zenodo Data Repository at doi:10.5281/zenodo.10245595 [76].
